# Echocardiographic Characteristics of Patients with Multiple Acute Concomitant Cerebral Infarcts

**DOI:** 10.3390/jcm14248969

**Published:** 2025-12-18

**Authors:** Aviya R. Jacobs, David Leibowitz, Naaem Simaan, Issa Metanis, Hamza Jubran, Fatma Shalabi, Tamer Jubeh, Ronen R. Leker

**Affiliations:** 1 Department of Medicine, Hadassah-Hebrew University Medical Center, Jerusalem 91120, Israel; aviya.jacobs@mail.huji.ac.il; 2 Department of Cardiology, Hadassah-Hebrew University Medical Center, Jerusalem 91120, Israel; oleibo@hadassah.org.il; 3 Department of Neurology, Ziv Medical Center, Safed 13100, Israel; naaem.simaan@gmail.com; 4 Azrieli Faculty of Medicine, Bar Ilan University, Safed 13100, Israel; 5 Stroke Unit, Department of Neurology, Hadassah-Hebrew University Medical Center, Jerusalem 91120, Israel; issa.meta@yahoo.com (I.M.); hamzaj@hadassah.org.il (H.J.); fatma.shalabi@gmail.com (F.S.); tamer00001@hotmail.com (T.J.)

**Keywords:** stroke, cerebral infarction, cardiac embolism, shower of emboli, cardiomyopathy, echocardiography

## Abstract

**Background/Objectives**: Only limited data on the characteristics and outcomes of patients with multiple acute concomitant cerebral infarcts (MACCI) exist. MACCI may imply a cardioembolic source and echocardiography is important in evaluating for potential embolic sources. However, data comparing echocardiographic features in MACCI to those observed in patients with single presumed embolic cerebral infarctions (SACI) are lacking. Thus, we aim to compare echocardiographic features between MACCI and SACI patients. **Methods**: We retrospectively analyzed data from a prospective stroke registry. The diagnosis of stroke secondary to MACCI and SACI was confirmed by MRI. Data on echocardiographic features, demographics, medical history, and functional status were extracted and compared between the groups. **Results**: Overall, 145 patients were included (83 SACI and 62 MACCI). MACCI patients were significantly older (mean ± sd 68.08 ± 13.04 vs. 62.70 ± 14.18; *p* = 0.021) and had higher rates of diabetes (35% vs. 25%; *p* = 0.014) and prior strokes (15% vs. 8%; *p* = 0.032). The only echocardiographic parameter that differed between the groups was left ventricular mass index (LVMI), which was significantly higher in the MACCI group after adjusting for age (aOR 1.02, 95% CI [1,1.04]; *p* = 0.042). MACCI was associated with higher mortality rates (34.4% vs. 18.1%, *p* = 0.041). No correlation was found between LVMI and stroke severity or outcomes. **Conclusions**: LVMI was significantly higher in MACCI patients, possibly reflecting undiagnosed hypertension, cardiomyopathy or systemic disease as potential thromboembolic mechanisms responsible for stroke. Larger studies are needed to further assess its potential role in the pathology of MACCI.

## 1. Introduction

Ischemic stroke is the leading cause of severe long-term disability and is currently one of the largest contributors to mortality worldwide [1]. Up to one-third of stroke cases are classified as being due to an unidentified cause. Cardiac embolism (CE) accounts for up to 30% of all strokes and is most frequently secondary to atrial fibrillation. CE-associated strokes tend to be more severe and are linked to higher mortality rates and elevated risk of early and long-term recurrence compared to other forms of stroke [2]. The diagnosis of a CE stroke relies on the identification of a potential cardiac source in the absence of significant cerebrovascular atherosclerotic occlusive disease or other identifiable causes of stroke such as arterial dissection, vasculitis and hypercoagulability states [3]. Causes of CE disorders include atrial fibrillation (AF), left ventricle (LV) thrombus, patent foramen ovale (PFO), LV and left atrial (LA) dysfunction, valvular heart disease, cardiac tumors and structural heart defects, among others. Echocardiography is vital in the diagnosis of these conditions and is therefore a crucial part of the evaluation, diagnosis and management of patients with suspected CE stroke. Among patients with CE stroke, one subgroup presents with multiple arterial-territory cerebral infarction (MACCI) defined as acute ischemic lesions in at least two arterial cerebral territories [4,5]. In a previous analysis, MACCI was associated with cardioembolic diseases, aortic arch atheroma, hematologic causes and vasculitis, but the etiology remained unidentified in 26% of the patients [6].

The term embolic stroke of undetermined source (ESUS) is used to describe a cryptogenic stroke with non-lacunar ischemic stroke on imaging and no confirmed etiology following adequate investigation for an embolic stroke mechanism [2,7]. In a prospective stroke cohort up to 10% of the CE or ESUS stroke patients were diagnosed with MACCI on MRI, with estimates ranging between 9 and 24% of all ischemic strokes [6,8].

Other cardiac disorders including LV systolic dysfunction and cardiomyopathy increase the risk of thromboembolism and ischemic stroke [9,10].

Unfavorable short-term outcomes and in-hospital complications are more frequent in MACCI patients compared to those with a single acute cerebral infract (SACI) [11]. Therefore, it seems plausible that early identification of MACCI and its cause may enable early initiation of cause-specific preventive therapy and may prevent further strokes and reduce disability. Among patients with suspected CE stroke the initial post-stroke workup includes cardiac arrhythmia monitoring as well as cardiac and large vessel imaging. Multimodal cardiac imaging can diagnose LV ejection fraction (EF), LV wall motion abnormalities, PFO, LV diastolic dysfunction and LV thrombus, all of which have been associated with CE stroke [12,13,14,15,16]. However, data comparing echocardiographic features in MACCI patients to patients with SACI are lacking. Such data could be vital for identification, treatment and prevention of future stroke in MACCI patients. Accordingly, the aim of this study was to compare echocardiographic features between MACCI and SACI patients.

## 2. Materials and Methods

This study was a retrospective analysis of patients with CE stroke identified from a prospective registry of stroke patients admitted to Hadassah-Hebrew University Medical Center in Jerusalem, Israel. The study adhered to the ethical principles of the 1975 Helsinki Declaration. This study was conducted in accordance with and was authorized by the local ethics committee at Hadassah Hebrew University Hospital (approval code HMO-0713-20, 27 April 2020).

All individuals with a MACCI diagnosis treated during the period from January 2015 through December 2021 were eligible for inclusion. Eligible patients had undergone MRI studies including diffusion-weighted imaging (DWI) at b0, b500, and b1000, an adjusted diffusion coefficient (ADC), and fluid-attenuated inversion recovery (FLAIR) sequences, as previously described [5]. Patients were excluded if MRI could not be performed due to safety or tolerance limitations, including implanted metallic fragments, cardiac pacing devices or severe claustrophobia.

**Figure 1:**
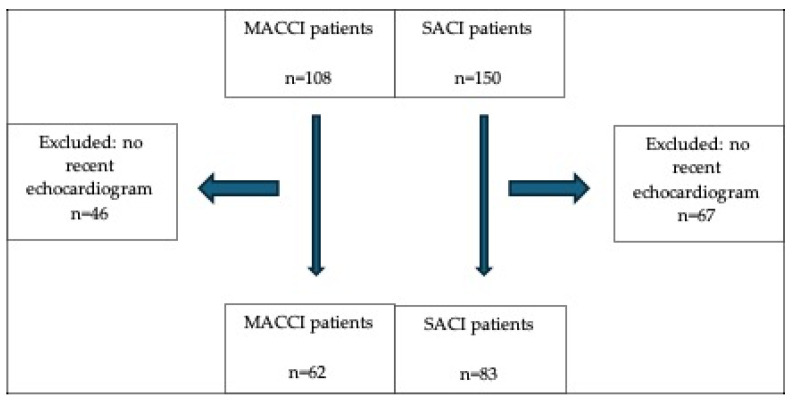
Study design.

Furthermore, CT angiography was performed in all included patients unless the use of iodinated contrast posed an unacceptable risk, including cases of contrast allergy or renal dysfunction. As previously described, MACCI was defined by the presence of multiple acute cerebral infarcts detected on DWI MRI, distributed across distinct vascular territories and lacking a single unifying arterial source [5]. Consecutive patients diagnosed with SACI from the same prospective institutional database and study period, who had completed an inpatient MRI and a full outpatient follow-up, were randomly selected and included as controls at a 1:1.5 ratio. Controls were not matched by any prespecified criteria and were consecutively selected from the database. Only patients who had an echocardiogram within 3 months of the index stroke as part of the post-stroke workup, were included (Figure 1). Transthoracic echocardiography took place within the Hadassah-Hebrew University Medical Center in Jerusalem, Israel. All patients had standard 2-D and Doppler echocardiography according to the recommendations of the European Association of Cardio-vascular Imaging [17]. In our institution patients undergoing echocardiography to determine possible cardiac source of emboli routinely undergo color Doppler as well as agitated saline injection to test for the presence of a PFO. Transesophageal echocardiography was not routinely performed in our patient population. Measurements were extracted from the official test results, after an echocardiographic specialist’s review.

The relevant data was extracted from the hospital files including baseline characteristics and comorbidities (age, sex, risk factors, clinical presentation, and diagnosis) and echocardiographic variables including indices of ventricular structure and systolic/diastolic function, left atrial size, LV mass, presence of PFO, valve insufficiency and wall motion dysfunction [18]. We included patients with AF as indicated in their history, as such data regarding the chronicity of the diagnosis was not disclosed.

Stroke severity was measured with the NIH stroke scale (NIHSS) [19] and outcomes were determined at 90 days with the modified Rankin Score (mRS) [20,21] assessed upon admission, discharge and at 90 days follow up.

In addition, we analyzed the echocardiographic features within the MACCI group in relation to the control group of SACI patients, with a sub-analysis of the stroke severity based on the NIHSS and mRS on admission and 3 months later.

### Statistical Evaluation

Statistical analyses were performed using R 4.4.2 (R Foundation for Statistical Computing, Vienna, Austria). Continuous data is presented with mean and SD or median and IQR (inter-quartile range) for normally and non-normally distributed data, respectively. All *p*-values were two tailed, and *p* < 0.05 was considered significant.

Differences between groups were studied using *t*-test or Wilcoxon test, as appropriate. Categorical and binary variables are presented with count and percent and differences between the groups were performed using chi-squared or Fisher’s exact test, as appropriate.

The echocardiographic measurement of interest was further analyzed using a logistic regression, to test their effect on the type of stroke, after adjusting for basic baseline characteristics of age, history of hypertension and history of diabetes. Each adjustment was tested individually. Additionally, a logistic regression was implemented, adding all baseline characteristics (age, history of hypertension and history of diabetes), as well as echocardiographic variables showing a *p* value of <0.2 in their association with stroke types. A similar linear regression was performed for the effect of LV mass index and age on NIHSS and mRS scores and delta in scores from admission to three months.

The association between stroke and mortality was examined using Kaplan–Meier survival curves, long-rank tests and cox proportional hazard model.

## 3. Results

### 3.1. Demographic Data and Clinical Characteristics

The study population included 108 patients with MACCI and 150 with SACI. Of those, 62 patients with MACCI and 83 with SACI had echocardiographic exams and were included in the current analysis (Figure 1). The demographic and baseline clinical characteristics are presented in Table 1. Patients with MACCI were significantly older (mean ± sd 68.08 ± 13.04 years and 62.70 ± 14.18; *p* = 0.021). Patients with MACCI were more likely to suffer from diabetes (52% vs. 30%, *p* = 0.014) and past strokes (24% vs. 10%, *p* = 0.032). Other risk factors for stroke including presence of malignancy, hypertension, atrial fibrillation, hyperlipidemia and congestive heart failure were numerically more frequent in patients with MACCI but the difference between the groups did not reach statistical significance (Table 1).

### 3.2. Comparison of Echocardiography Variables

The comparison of echocardiographic variables between patients with MACCI vs. SACI is presented in Table 2. The only echocardiographic feature that significantly differed between the groups was the LV mass index (LVMI), which was higher in the MACCI group (94.38 ± 32.91 g/m^2^ and 81.71 ± 21.18 g/m^2^, *p* = 0.025). There were no significant differences in regional wall abnormalities, diastolic function, valve insufficiency, EF, or presence of PFO (Table 2). The association of LVMI with MACCI remained significant after adjusting for age, presence of hypertension and diabetes (*p* = 0.02 for all; Table 3). Subgroup analysis of hypertrophy degree based on LVMI and sex as presented in Table S1. LVMI was stratified into degrees of hypertrophy (normal, mild, moderate and severe) based on gender-specific European Association of Cardio-vascular Imaging guidelines. The analysis revealed a statistically significant difference in the overall prevalence of left ventricular hypertrophy (LVH) between the study groups (*p* = 0.048). While only 14% of patients in the SACI group exhibited LVH, the prevalence in the MACCI group was markedly higher at 32%. Furthermore, the MACCI group demonstrated a trend toward more advanced disease, with a higher proportion of patients categorized as having severe hypertrophy compared to the SACI group.

There was no significant association between age and LVMI and the NIHSS and mRS scores upon admission, discharge and three months post-stroke (Table 2 and Table 4).

### 3.3. Comparison of Long-Term Outcomes in MACCI vs. SACI Patients

**Figure 2:**
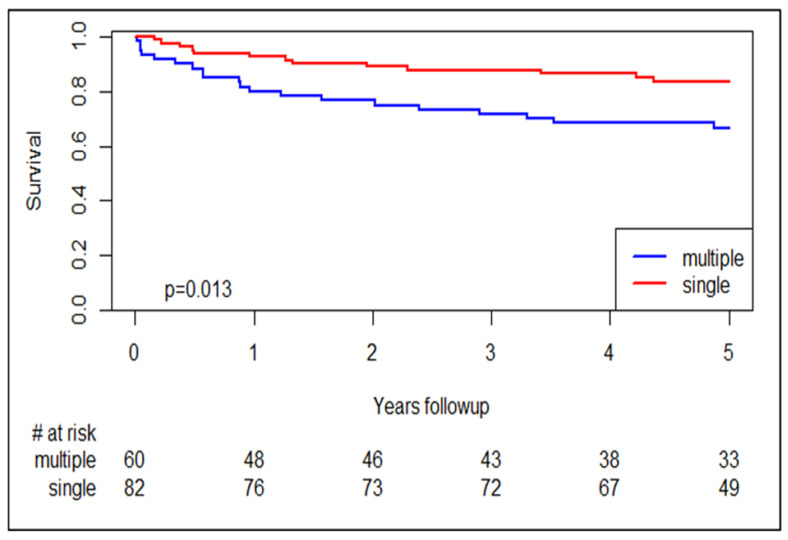
Kaplan-Maier 5-year survival, MACCI vs. SACI. # = the number of patients at risk.

The median follow-up period was seven years. During follow-up, 25% of the patients died with significantly higher mortality rates observed among MACCI patients (34.4% vs. 18.1%, *p* = 0.041). Kaplan–Mayer survival curves showed that the difference in survival between the MACCI vs. SACI patients was statistically significant, (Figure 2, *p* = 0.013). However, in a cox proportional hazard model, LVMI was not associated with survival (hazard ratio 1.011 95% CI 0.997–1.025, *p* = 0.141.

## 4. Discussion

In this study, we compared echocardiographic variables between patients diagnosed with MACCI and SACI. The main novel finding of the current study is that the LVMI was significantly higher in the MACCI group. However, LVMI was not associated with stroke severity on presentation, functional disability, rates of recurrent stroke or survival.

In prospective stroke cohorts 9–24% of all ischemic stroke patients are diagnosed with MACCI on MRI [6,8]. CE and especially ESUS echocardiographic biomarkers are a growing interest in recent research given the unmet clinical need to identify patients who may be predisposed to ESUS [22,23,24,25]. The main echocardiographic features examined in previous studies included EF, regional wall motion abnormality, LA enlargement, LVMI, LA spheric modeling, LA volume index, atrial stiffness, diastolic function and LA rotational flow [12]. The focus on these features specifically is based on the theory that they all may be related to the presence of cardiopathy, which in turn can lead to thromboembolic events.

However, the existing data remains controversial [26]. For instance, Ramasamy et al. [12], did not find an association between LV EF or LV wall motion abnormality and ESUS in agreement with the current results. However, after excluding ESUS patients with any evidence of ipsilateral carotid atherosclerosis, including those with under 50% stenosis, lower LVEF appeared to be associated with ESUS.

In the current study, the LVMI was the only statistically significant echocardiographic feature associated with MACCI, regardless of age or presence of diabetes mellitus or hypertension. Our findings are supported by those of Boyd et al. [27], who compared AF patients with and without LA thrombus. The strongest predictor of left atrial appendage thrombus was LV mass, and although the reported history of hypertension was not statistically significant, systolic blood pressure was a predictor of thrombus presence, suggesting there may be a higher incidence of undiagnosed hypertension in AF patients who had an atrial thrombus.

A study by Gąsiorek et al. [15] included ESUS patients under the age of 65, comparing their echocardiographic features among other biomarkers. The study found a significant association between increased arterial stiffness and indices of diastolic dysfunction and ESUS, one of the statistically significant markers being LVMI. A possible explanation is that the incidence of undiagnosed hypertension is higher in younger patients, which could in turn explain the higher evidence of LVMI in this group. Another possible explanation for the positive association between MACCI and LVMI could be that LVMI is an indicator of atrial or ventricular cardiomyopathy [15,22,28]. This explanation aligns with our subgroup analysis, which indicates a higher overall prevalence of LVH in the MACCI group compared with the SACI group, a difference that was statistically significant. Ferkh et al. [22], found that alterations in LA shape, especially increased spheric remodeling, are present in ESUS, AF and CE patients and that this may be an early marker of LA myopathy. This contrasts with the findings of Pires et al. [28], who could not show a significant association between atrial cardiopathy, defined by LV and LA basic measurements on echocardiography and electrocardiogram, and ESUS. Interestingly, Liu et al. [29], found that atrial cardiomyopathy was significantly associated with a poor functional outcome in non-cardioembolic stroke patients.

Further hypotheses as to the connection between MACCI and LVMI, could be that elevated LVMI is associated with a systemic disorder such as vasculitis, amyloidosis or Fabry disease [30,31,32,33]. Knight et al. [30], identified functional and structural cardiac abnormalities associated with different levels of amyloidosis burden, using cardiac magnetic resonance and echocardiography. LVMI had a high probability of being abnormally high even at low cardiac amyloid burden. Oyamada et al. [31] found that patients diagnosed with Kawasaki disease demonstrated a higher LVMI compared to controls. These data suggest that patients with abnormal diastolic function and increased arterial stiffness, which may be associated with elevated LVMI, may have a higher chance of incurring MACCI.

The current results suggest a significant difference in LVMI between MACCI and SACI, which suggests that undiagnosed hypertension may play a role in the thromboembolic mechanism in these patients.

Regarding outcome, our analysis shows a significant increase in mortality among patients with MACCI compared to SACI controls, confirming similar data from previous studies [2,8,11]. This is in line with the findings that patients with MACCI were older and more often had risk factors for stroke as well as overt cardiovascular disease. They were more frequently treated with ACE inhibitors and statins, also reflecting their higher comorbidity burden and increased risk for stroke. However, we could not confirm a correlation between the LVMI and initial stroke severity, functional outcomes including independence or significant disability, long-term stroke recurrence or survival. Therefore, the importance of identifying a high LVMI in a patient with a suspected CE stroke remains unclear. However, since MACCI is known to cause a more severe clinical presentation and cause a more significant long-term disability, it would stand to reason that echocardiography could be used to identify patients who could benefit from more robust hypertension investigation and control [5]. Whether the mere presence of high LVMI should drive physicians to perform an MRI in the setting of an acute suspected CE stroke for a possible diagnosis of MACCI in an effort to start more efficient preventive therapy earlier will necessitate further study.

Strengths of the study include the prospective patient enrollment and the relatively large number of patients with MACCI [5]. Adding a comparison of echocardiographic findings to the question of suspected CE origin is a novel approach, not previously addressed [34].

Our study has several limitations. First, it was retrospective in nature and thus liable to bias. Second, the study was conducted in a single tertiary academic center, which may limit the generalizability of the results. Third, we could only include a relatively small number of patients, and therefore our results should be interpreted with caution. Fourth, we could not ascertain compliance with treatment due to the retrospective nature of the study and hence we could not include specific drug use into the regression models. Fifth, we only used atrial size in the anteroposterior plane. It is possible that measurements in different places could have added important information to the data presented but these were not available to us as the routine echocardiographic tests completed only use the anteroposterior plane for such measurements at our institution. Finally, transesophageal echocardiography was not routinely performed in the population. Routine performance of transesophageal echocardiography could have led to identification of relatively rare conditions such as papillary fibroelastoma which may have been missed by routine trans-thoracic echocardiography.

## 5. Conclusions

In conclusion, MACCI is associated with a more severe clinical presentation and increased rates of severe long-term disability and mortality. When comparing echocardiographic features between MACCI and control group, we found LVMI to be independently and significantly higher in the MACCI group possibly reflecting the fact that MACCI patients represent an older at-risk population for cerebrovascular disease. In this context, LVMI can be viewed as a potential marker for more advanced cardiovascular disease. However, LVMI was not associated with initial stroke severity, functional outcomes or survival. Nevertheless, these findings could have important clinical implications in MACCI patients. Future larger-scale studies are needed to understand the relevance of this finding, especially regarding undiagnosed hypertension.

## Figures and Tables

**Table 1:** Baseline characteristics of the study population.

	MACCI *n* = 62	SACI *n* = 83	*p*
Age (mean ± sd)	68.08 ± 13.04	62.70 ± 14.18	0.021
Gender (male, %)	31 (50.0)	47 (56.6)	0.533
BSA (mean ± sd)	1.79 ± 0.24	1.87 ± 0.25	0.129
HTN (%)	44 (71.0)	53 (63.9)	0.470
AF (%)	12 (19.4)	10 (12.0)	0.327
DM (%)	32 (51.6)	25 (30.1)	0.014
IHD (%)	16 (25.8)	30 (36.1)	0.253
LIPIDS (%)	29 (46.8)	35 (42.2)	0.701
CHF (%)	11 (17.7)	13 (15.7)	0.914
CRF (%)	8 (12.9)	4 (4.8)	0.126
Old stroke (%)	15 (24.2)	8 (9.6)	0.032
Valve disease (%)	9 (14.5)	4 (4.9)	0.075
Statins (%)	29 (46.8)	8 (9.6)	<0.001
Anti-PLT (%)	34 (55.7)	28 (33.7)	0.014
Coumadin (%)	4 (6.5)	0 (0.0)	0.067
ACEi (%)	16 (25.8)	1 (1.2)	<0.001
NOACS (%)	2 (3.2)	2 (2.4)	1.000
Smoking (%)	12 (19.4)	28 (33.7)	0.073
Cancer (%)	16 (25.8)	10 (12.0)	0.055
admission NIHSS (median [IQR])	5 [3, 7]	4 [2, 8]	0.487

MACCI = multiple arterial-territory cerebral infarction, SACI = single arterial-territory cerebral infarction, BSA = Body Surface area, HTN = Hypertension, AF = Atrial Fibrillation, DM = Diabetes Miletus, IHD = Ischemic Heart Disease, CHF = Congestive Heart Failure, CRF = Chronic Renal Failure, Anti-PLT = Anti-platelet therapy, ACEi = Angiotensin Converting Enzyme inhibitor, NOACS = New Oral Anticoagulants. NIHSS = National Institutes of Health Stroke Scale.

**Table 2:** Comparison of echocardiography variables.

	MACCI *n* = 62	SACI *n* = 83	*p*
LVMI (mean ± sd)	94.38 ± 32.91	81.71 ± 21.18	0.025
MV Ee’ lateral (median [IQR])	9.79 [6.25, 12.19]	8.51 [6.66, 11.53]	0.528
MV Ee’ septal (median [IQR])	13.90 [11.37, 16.65]	6.97 [5.79, 11.37]	0.225
Regional wall (%)	16 (26.7)	12 (14.5)	0.109
LA (mean ± sd)	38.90 (7.28)	37.94 (5.92)	0.384
TR gradient (mean ± sd)	30.17 (13.37)	27.14 (10.84)	0.184
Ascending aorta (mean ± sd)	32.88 (4.31)	32.57 (4.73)	0.692
Ascending aorta indexed to BSA (mean ± sd)	18.97 (2.9)	17.85 (3.17)	0.081
EF (%)			0.652
Normal	51 (82.3)	72 (86.7)	
Mild-moderate	9 (14.5)	10 (12.0)	
Severe	2 (3.2)	1 (1.2)	
AR (%)			0.246
Normal	50 (80.6)	66 (79.5)	
Mild-moderate	8 (12.9)	15 (18.1)	
Severe	0 (0.0)	1 (1.2)	
MR (%)			0.801
Normal	39 (62.9)	57 (68.7)	
Mild-moderate	20 (32.3)	24 (28.9)	
Severe	1 (1.6)	1 (1.2)	
PFO (%)	5 (13.9)	8 (11.6)	0.789

MACCI = multiple arterial-territory cerebral infarction, SACI = single arterial-territory cerebral infarction, LVMI = Left Ventricular Mass Index, MV Ee’ = Mitral Valve Early Diastolic Velocity (E)/Lateral Mitral Annulus Early Diastolic Velocity (e’), LA = Left Atrium, TR gradient = Tricuspid Regurgitation gradient, BSA = Body Surface Area, EF = Ejection Fraction, AR = Aortic Regurgitation, MR = Mitral Regurgitation, PFO = Patent Foramen Ovale.

**Table 3:** Multivariate analysis to predict MACCI vs. SACI.

	OR	95% CI	*p*
Age	1.05	1–1.11	0.0402
HTN	1.09	0.33–3.63	0.8822
DM	1.24	0.38–4.08	0.7174
LVMI	1.02	1–1.05	0.0204
Regional wall	1.03	0.28–3.86	0.9672
TR gradient	0.99	0.95–1.05	0.8023

MACCI = multiple arterial-territory cerebral infarction, SACI = single arterial-territory cerebral infarction, HTN = Hypertension, DM = Diabetes Miletus, LVMI = Left Ventricular Mass Index, TR = Tricuspid regurgitation.

**Table 4:** Outcomes in patients with MACCI and SACI.

	MACCI *n* = 62	SACI *n* = 83	*p*
Discharge NIHSS (median [IQR])	3.00 [2.00, 6.00]	3.00 [1.00, 4.00]	0.108
90 days NIHSS (median [IQR])	2.00 [1.00, 4.00]	2.00 [1.00, 4.00]	0.407
Discharge mRS (median [IQR])	2.00 [1.00, 3.00]	1.00 [1.00, 2.00]	0.007
90 days mRS (median [IQR])	2.00 [1.00, 3.00]	1.00 [1.00, 2.00]	<0.001
Recurrent stroke (%)	10 (21.3)	21 (28.4)	0.510
MI (%)	2 (4.3)	2 (2.7)	0.641
Cardiac hospitalization (%)	6 (12.8)	9 (12.2)	1.000
Survival (%)	40 (65.6)	68 (81.9)	0.041

MACCI = multiple arterial-territory cerebral infarction, SACI = single arterial-territory cerebral infarction, NIHSS = National Institutes of Health Stroke Scale, mRS = Modified Rankin Scale, MI = myocardial infraction.

## Data Availability

The original contributions presented in this study are included in the article/Supplementary Materials. Further inquiries can be directed to the corresponding author.

## Supplementary Materials

The following supporting information can be downloaded at: https://www.mdpi.com/article/10.3390/jcm14248969/s1, Table S1: Subgroup analysis of hypertrophy degree and sex, based on LVMI.

## Abbreviations

The following abbreviations are used in this manuscript:

MACCI	multiple acute concomitant cerebral infarcts
SACI	single presumed embolic cerebral infarctions
LVMI	left ventricular mass index
CE	Cardiac embolism
AF	atrial fibrillation
LV	left ventricle
PFO	patent foramen ovale
LA	left atrial
ESUS	embolic stroke of undetermined source
EF	ejection fraction
DWI	diffusion-weighted imaging
ADC	adjusted diffusion coefficient
FLAIR	fluid-attenuated inversion recovery
NIHSS	NIH stroke scale
mRS	modified Rankin Score
LVH	Left Ventricular Hypertrophy
