# Supplementary material for: Echocardiographic Characteristics of Patients with Multiple Acute Concomitant Cerebral Infarcts
Source: J Clin Med. 2025 Dec 18;14(24):8969. doi: 10.3390/jcm14248969 (PMC12734069; doi:10.3390/jcm14248969)
Supplement: Supplementary file 1 [file jcm-14-08969-s001.zip › jcm-4010622-supplementary.pdf]

Supplementary Materials:

Table S1. Subgroup analysis of hypertrophy degree, based on LVMI and sex.

	**MACCI**	**SACI**	***p***
Hypertrophy %			0.048
Normal	68	86	
Mild LVH	13	7	
Moderate LVH	3	2	
Severe LVH	16	5	

MACCI = multiple arterial-territory cerebral infarction, SACI = single arterial-territory cerebral infarction, LVH = Left ventricular hypertrophy.
